# Cytotoxic Potential of Biogenic Zinc Oxide Nanoparticles Synthesized From *Swertia chirayita* Leaf Extract on Colorectal Cancer Cells

**DOI:** 10.3389/fbioe.2021.788527

**Published:** 2021-12-15

**Authors:** Hadgu Mendefro Berehu, Anupriya S, Md Imran Khan, Rajasree Chakraborty, Kousalya Lavudi, Josthna Penchalaneni, Bibhashee Mohapatra, Amrita Mishra, Srinivas Patnaik

**Affiliations:** ^1^ Disease Biology Laboratory, School of Biotechnology KIIT Deemed to Be University, Odisha, India; ^2^ Department of Biotechnology, Sri Padmavati Mahila Visvavidyalam, Tirupati, India

**Keywords:** biogenic, colorectal cancer, zinc oxide nanoparticles, *Swertia chirayita*, cytotoxicity

## Abstract

Chemotherapy side effects, medication resistance, and tumor metastasis impede the advancement of cancer treatments, resulting in a poor prognosis for cancer patients. In the last decade, nanoparticles (NPs) have emerged as a promising drug delivery system. *Swertia chirayita* has long been used as a treatment option to treat a variety of ailments. Zinc oxide nanoparticles (ZnO-NPs) were synthesized from ethanolic and methanolic extract of *S. chirayita* leaves. ZnO-NPs were characterized using UV-visible spectroscopy, Fourier transform infrared spectroscopy (FTIR), scanning electron Microscopy (SEM), high-resolution transmission electron microscopy (HRTEM), and X-ray diffraction (XRD). Its anti-cancer activities were analyzed using cytotoxicity assays [MTT assay and acridine orange (AO) staining] and quantitative real-time PCR (qRT-PCR) using colorectal cancer (CRC) cells (HCT-116 and Caco-2) and control cells (HEK-293). The ZnO-NPs synthesized from the ethanolic extract of *S. chirayita* have an average size of 24.67 nm, whereas those from methanolic extract have an average size of 22.95 nm with a spherical shape. MTT assay showed NPs’ cytotoxic potential on cancer cells (HCT-116 and Caco-2) when compared to control cells (HEK-293). The IC_50_ values of ethanolic and methanolic extract ZnO-NPs for HCT-116, Caco-2, and HEK-293 were 34.356 ± 2.71 and 32.856 ± 2.99 μg/ml, 52.15 ± 8.23 and 63.1 ± 12.09 μg/ml, and 582.84 ± 5.26 and 615.35 ± 4.74 μg/ml, respectively. Acridine orange staining confirmed the ability of ZnO-NPs to induce apoptosis. qRT-PCR analysis revealed significantly enhanced expression of E-cadherin whereas a reduced expression of vimentin and CDK-1. Altogether, these results suggested anti-cancer properties of synthesized ZnO-NPs in CRC.

## 1 Introduction

Colorectal cancer (CRC) is the second most fatal cancer and the third most common malignant tumor in the globe. In 2018, 1.8 million new CRC cases were recorded, with around 900,000 fatalities (Bray et al., 2018), and the number of new cases is expected to rise to nearly 2.5 million by 2035 (Dekker et al., 2019). Adjuvant radiotherapy and chemotherapy, together with surgery, help extend the patient’s life. However, the abovementioned common treatments have drawbacks, including drug resistance and adverse effects like fatigue, alopecia, anemia, etc. due to non-specific distribution of drugs (Banerjee et al., 2017; Van der Jeught et al., 2018). These difficulties pushed scientists to explore different approaches for cancer therapy with fewer side effects. The use of nanotechnology-based techniques in cancer therapy has yielded encouraging outputs. As a result, possible treatment alternatives that are efficient, affordable, and have excellent biocompatibility must be sought.

In the recent few decades, nanotechnology’s implications have progressed to a higher level. Various forms of organic and inorganic nanoparticles, particularly metal nanoparticles and their composites, have been investigated extensively and used in a variety of applications (Verma et al., 2018a, 2016). Metal oxide nanoparticles have shown to have potential and a broad range of applications in the biomedical area, including anticancer drug/gene delivery, cell imaging, and biosensing (Khalil et al., 2017; Mishra et al., 2017; Verma et al., 2018b). Zinc is well regarded as an essential trace element that may be found in all human tissues, such as the brain, muscle, bone, and skin. Zinc participates in metabolic activities and has an essential role in nucleic acid and protein synthesis, neurogenesis, hematopoiesis, and as a major cofactor of different enzyme systems (Ruszkiewicz et al., 2017). Zinc oxide nanoparticles (ZnO-NPs) have distinctive physical and chemical properties, due to their broad bandgap and high exciton binding energy. It is one of the most significant metal oxide nanoparticles and is being widely used in a variety of disciplines (Ruszkiewicz et al., 2017).

Nanoparticles have entirely unique properties since they have distinct features such as size, shape, and distribution. There are several procedures available for the fabrication of nanoparticles, including chemical-mediated, radiation-mediated, and electrochemical- and microwave-assisted processes, as well as green approaches (Gan and Li, 2012; Kumari et al., 2019). Biogenic metallic nanoparticles are highly water-soluble, biocompatible, and less toxic. Several studies have shown that various plant extracts are used to synthesize ZnO-NPs; for instance, aqueous extract of *Deverra tortuosa* (Selim et al., 2020), leaf extracts of *Cassia fistula* and *Melia azedarach* (Naseer et al., 2020), *Cassia auriculata* flower extract (Seshadri et al. 2021), and *Hibiscus rosa-sinensis* leaf extract (Divya et al., 2013) were employed as reducing agents for zinc nitrate to fabricate ZnO-NPs.

*Swertia chirayita*, a centuries-old plant, is a perennial herb endemic to the temperate Himalayan area. Because of the annual/biennial plant found in Nepalese woods, it is often referred to as Nepali neem (Aleem and Kabir, 2018). This herb or shrub grows up to a height of 1.5 m in the sub-temperate area of the Himalaya. In India, 40 species of *Swertia* have been registered so far (Kumar and Van Staden, 2016). *S. chirayita* is well-known for its bitter flavor and has a long history of usage in traditional medicine. It has an antimicrobial effect that works against both Gram-positive and Gram-negative bacteria. According to the Unani lists, different parts of the plant are applied as medicine for different disorders such as liver and cardiac diseases, cough, scanty urine, melancholia, dropsy, sciatica, and skin diseases. It is also used as a tonic in gastrointestinal illnesses including dyspepsia/anorexia, constipation, anti-malaria (anti-fever), bronchial asthma, etc. (Khanal et al., 2014).

Ahmad et al. (2021) reported that both crude and purified *S. chirayita* extracts significantly reduced cell growth and induced apoptosis, indicating that it had anticancer activities. *S. chirayita* is a key ingredient in several Ayurvedic medications and is listed in the British Herbal Pharmacopoeia in 1983. Secoiridoid glucosides, swertiamarin, sweroside, gentiopicroside, amarogentin, and amaroswerin, as well as several tetrahydroxyxanthone derivatives, are the major compounds present in *S. chirayita* (Wawrosch, 2005).

In this study, we synthesized ZnO-NPs from ethanol and methanol extracts of *S. chirayita* leaves as capping and reducing agents, while zinc nitrate hexahydrate serves as precursors of zinc oxide nanoparticles. The formation of ZnO-NPs was verified and characterized by various microscopic and spectroscopic tests that involve UV-visible spectroscopy (UV-vis), dynamic light scattering (DLS), Fourier transform infrared spectroscopy (FTIR), X-ray diffraction (XRD), scanning electron microscopy (SEM), and transmission electron microscopy (TEM). The main objective of this study was to show the cytotoxic activities of biogenic ZnO-NPs from ethanol and methanol extracts of *S. chirayita* leaves against two CRC cell lines, HCT-116 and Caco-2, and HEK-293 as control. To our knowledge, this is the first report on ethanol and methanol extracts of *S. chirayita* mediating the synthesis of ZnO-NPs. Therefore, this study will give alternative therapeutic approaches to use plant-based synthesized nanoparticles that are eco-friendly and cost-effective/affordable.

## 2 Materials and Methods

### 2.1 Chemicals and Instruments

Zinc nitrate hexahydrate [Zn (NO_3_)_2_·6H_2_O] was purchased from Sigma-Aldrich, United States (CAS #: 10196-18-6); methanol (CAS #: 67-56-1) and acridine orange (CAS#: 10,127-02-3) were obtained from SRL; and ethanol (CAS #: 64-17-5) and sodium hydroxide (CAS#: 1310-73-2) were purchased from Merck. The following technologies were used to analyze quantitative and qualitative assessments: gas chromatography-mass spectrometry (GC-MS) (Agilent 7890A, United States), UV-visible spectroscopy (Cary 100 UV-Vis Spectrophotometer, United States), FTIR (100 Spectrum, Perkin Elmer, Germany), XRD (Rigaku Smart Lab 8A Series, Japan), high-resolution (HR)-TEM (HRTEM—JEOL JEM-2100 PLUS, United States), SEM (Carl ZEISS Supra 55, Germany), 96-well plate reader (Epoch microplate spectrophotometer, BioTek, Vermont, United States), heater-stirrer (IKA Magnetic Stirrers Hot Plate, RH digital white, India), rotary evaporator (4003 Heidolph, Germany), bench top fluorescence microscopy (FLoid™ Cell Imaging Station, United States), and centrifuge (5804/5804R Eppendorf benchtop centrifuge, United States).

### 2.2 Cell Lines and Cell Culture Preparation

Colorectal cancer cell lines (HCT-116 and Caco-2) and control cell line (HEK-293) were procured from the National Centre For Cell Science (NCCS), Pune, India. The cells were cultured in Dulbecco’s Modified Eagle’s Medium (DMEM) added with 10% heat-inactivated fetal bovine serum (FBS), 1% of l-glutamine, and 1% of antibiotics (penicillin/streptomycin) and incubated at 37°C in a humidified atmosphere (5% CO_2_).

### 2.3 Plant Extraction

Healthy leaves were selected and washed thoroughly with distilled water to get rid of any debris and then dried at room temperature for 5 days. Then, the leaves were ground into a fine powder using a grinder and sieved to get fine powder. The ethanolic and methanolic extracts were prepared as per previous extraction protocols (Hussain et al., 2019). The plant powder (1 g) was soaked in 100 ml of 80% ethanol and 90% methanol and kept in a hot water bath at 50°C for 3–4 h. Then, the extract was filtered using a 0.45-μm Whatman filter paper and stored at −20°C for further use.

### 2.4 Gas Chromatography-Mass Spectrometry Analysis

The Agilent 7890A Gas Chromatograph (Agilent Technologies, United States) was used to analyze *S. chirayita* leaf extracts using a fused silica column packed with Elite-5MS capillary column (30 m in length, 250 m in diameter, and 0.25 m in thickness). The carrier gas was pure helium gas (99.99%) at a pressure of 7.6522 psi and a constant flow rate of 1 ml/min with an average velocity of 36.445 cm/s throughout the 32-min running time. An electron ionization energy technique was used for GC-MS spectrum detection, with a high ionization energy of 70 eV (electron volts) and a scan period of 0.2 s with fragments ranging from 40 to 600 m/z.

The injector temperature was kept at 250°C, and the injection volume was 1 L (split ratio 10:1). The temperature in the column oven was set at 50°C for 1.3719 min, increased at 10°C each minute until it reached 250°C, and then elevated to 325°C for 32 min. Phyto-constituents present in the plant extract sample were analyzed by comparing their retention time, peak area, peak height, and mass spectral patterns to those in the National Institute of Standards and Technology (NIST) 08 Mass Spectral Library’s spectral database of authentic compounds.

### 2.5 Preparation of Green-Synthesized Zinc Oxide Nanoparticles

Zinc oxide nanoparticles were prepared as described by previous studies with minor modifications (Singh et al., 2011). Zinc nitrate hexahydrate was dissolved in 100 ml of 1 mg/ml plant extract into 250-ml flask and stirred at 70°C. An aqueous solution of 100 ml of 200 mM sodium hydroxide was added drop wise using a burette into the mixture and stirred constantly for 3 h, using a magnetic stirrer (70°C). Then, the solution was kept overnight to settle down. The light yellow-colored pellet was collected after discarding the supernatant. The pellet was then washed three times using deionized water and centrifuged at 10,000 rpm for 10 min. The resulting precipitates were then dried in a hot air oven overnight at 100°C to form a creamy paste of ZnO-NPs. Then, the dried ZnO-NPs were finely ground into powder using mortar and pestle.

### 2.6 Characterization of Green-Synthesized ZnO-NPs

The green-synthesized zinc oxide nanoparticles were initially analyzed to know the optical absorption using the Cary 100 UV-vis spectrophotometer within the range of 200–800 nm. To identify the functional and phyto-chemical compounds that are involved in the reduction and stability of the synthesized ZnO-NPs, FTIR spectroscopic analysis (FTIR, PerkinElmer, Germany) was used. Further X-ray diffraction analysis was used to validate the crystalline nature and size of the nanoparticles (X-ray diffractometer, Rigaku Smart Lab 8A Series, Japan), equipped with 40 kV/30 mA X-ray, 2*θ*/*θ* scanning mode, CuKα1-X radiation (λ = 1.5406 A°), and a fixed monochromator in the range of 20°–80°. The average size of the nanoparticles was calculated using Scherrer approximation (Dp = 0.9*λ*/*β*Cos*θ*) (Selim et al., 2020). The ZnO nanopowder was further processed and examined by HR-TEM (HRTEM—JEOL JEM-2100 PLUS, United States). Morphological evaluations were acquired by a scanning electron microscope (Carl ZEISS Supra 55, Oberkochen, Germany).

### 2.7 Cell Viability Assay

The MTT assay [3-(4,5-dimethylthiazol-2-yl)-2,5-diphenyl tetrazolium bromide assay] was done in a 96-well plate to assess the cytotoxicity of the plant extract and ZnO-NPs (Mosmann, 1983). Caco-2 and HCT-116 cell lines and control HEK-293 cells were grown to ∼70% confluence in respective media, and the cells were treated with various concentrations (5, 25, 75, 150, and 300 μg/ml) of crude extract and ZnO-NPs for 24 h. Then, 0.5 μg/ml MTT solution was added to each well and incubated at 37°C for 4 h in the dark before being replaced with 200 μl MTT solvent. After 15 min on a rocker, the optical density of the solution on the plate was measured at 570 nm with a plate reader (Epoch microplate spectrophotometer, BioTek, Vermont, United States). As a positive control, doxorubicin (57.418 and 63.036 µM HCT-116 and Caco-2 cell lines, respectively) was used (Siddiquah et al., 2018). The experiment was performed three times.

### 2.8 Acridine Orange Staining

The acridine orange (AO) staining method is used to monitor cell death. It is often used to identify acidic vesicular organelles (Lin et al., 2017). It binds into cells nucleic acids, causing double-stranded DNA to glow green and RNA or single-stranded DNA to fluoresce orange-red when observed with fluorescence microscope. The cytoplasm and nuclei glow green under AO labeling, but acidic compartments like lysosomes and auto-phagolysosomes fluoresce bright-red or orange-red (Hwang et al., 2011). Caco-2 cell lines were seeded in six-well plates and treated with crude extracts and their respective ZnO-conjugated NPs. AO is prepared according to the manufacturer’s protocol [Sisco Research Laboratories (SRL)]. The medium was removed, and the cells were washed with 1 ml of 1X PBS, added with 10 µl of 12 μg/ml AO working solution, and kept for 5 min in room temperature. Prior to observation, the cells were washed with 1 ml of 1X PBS for the second time, then observation was done by fluorescence microscopy (Lin et al., 2017). The proportion of cells with green and red fluorescence was determined using bench top fluorescence microscopy (Floid™ Cell Imaging Station, United States) at ×40 magnification with excitation wavelengths of 482/18 (green) and 586/15 (red).

### 2.9 Gene Expression by Quantitative Real-Time PCR

To examine effect of the crude ethanol extract and its ZnO-NPs on the expression level of cancer marker genes (CDK1, E-cadherin, and vimentin) in Caco-2 cell line, quantitative real-time PCR (qRT-PCR) was conducted. GAPDH was used as a housekeeping gene. TRIzol RNA Isolation Reagents (Invitrogen™ TRIzol™ Reagent) was used to extract RNA from treated Caco-2 cell lines. The process was carried out as per the manufacturer’s protocol. cDNA was synthesized from the purified RNA using the Verso cDNA Synthesis Kit (Thermo Fisher Scientific). The quantity of the primers, cDNA, and master mix was prepared according to the procedure given by DyNAmo ColorFlash SYBR Green qPCR Kit (Thermo Fisher Scientific). The Eppendorf Mastercycler Realplex ep gradient RT-PCR instrument was used to perform the qRT-PCR. The PCR setting was as follows: 95°C to activate the enzyme for 7 min, followed by 40 cycles of 15 s at 95°C (denaturation) and followed by 30 cycles at 55°C at 25 s and 72°C at 25 s (annealing and synthesis). Finally, a dissociation curve was constructed following the PCR run to validate and justify the results. Primer sequences have been provided in Supplementary Table S3.

### 2.10 Statistical Analysis

GraphPad PRISM 8 software was used to compute all of the data as a mean ± standard error, and Origin software was also used to generate the graphs. All the tests were carried out three times.

## 3 Results

### 3.1 GC-MS Analysis of Bioactive Molecule Extract of *S. chirayita* Leaves

**FIGURE 1:**
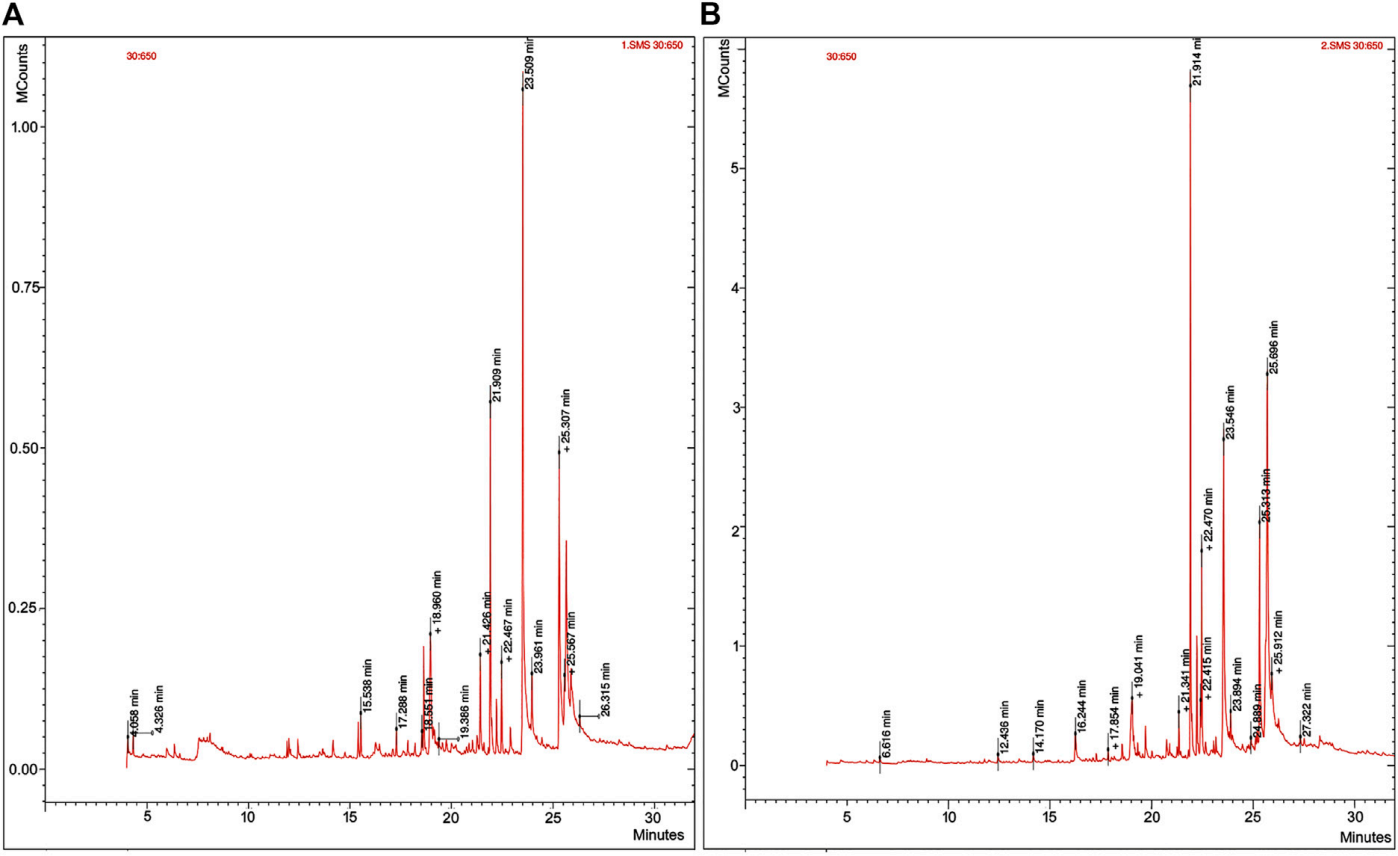
Gas chromatography-mass spectrometry (GC-MS) of *Swertia chirayita* leaf extracts. **(A)** Ethanol extract of *S. chirayita* leaves and **(B)** methanol extract of *S. chirayita* leaves.

GC-MS analysis of ethanol and methanol extracts of *S. chirayita* leaves recorded a total of 21 and 35 peaks, respectively. The observed results of the leaf extract of *S. chirayita* were in accordance with previous studies (Kumar and Van Staden, 2016; Li et al., 2017). The mass spectra were identified after comparison with the NIST library. The result indicated the presence of several phyto-compounds (Figure 1, Supplementary Tables S1 and 2).

### 3.2 Characterization of ZnO Nanoparticles Conjugated With *S. chirayita*

During the biosynthesis, the formation of ZnO-NPs was visualized. After 3 h of stirring at a hot plate, the color of the reaction solution gradually changed from white to light yellowish precipitate (Liu et al., 2020), and a change in the color of the solution during synthesis indicated that zinc nitrate had been reduced. These findings were consistent with prior studies of color changes in plant-based ZnO-NP production (Santhoshkumar et al., 2017). To verify the presence of the nanoparticles, microscopic and spectroscopic techniques were applied.

#### 3.2.1 UV-Visible Spectroscopy

**FIGURE 2:**
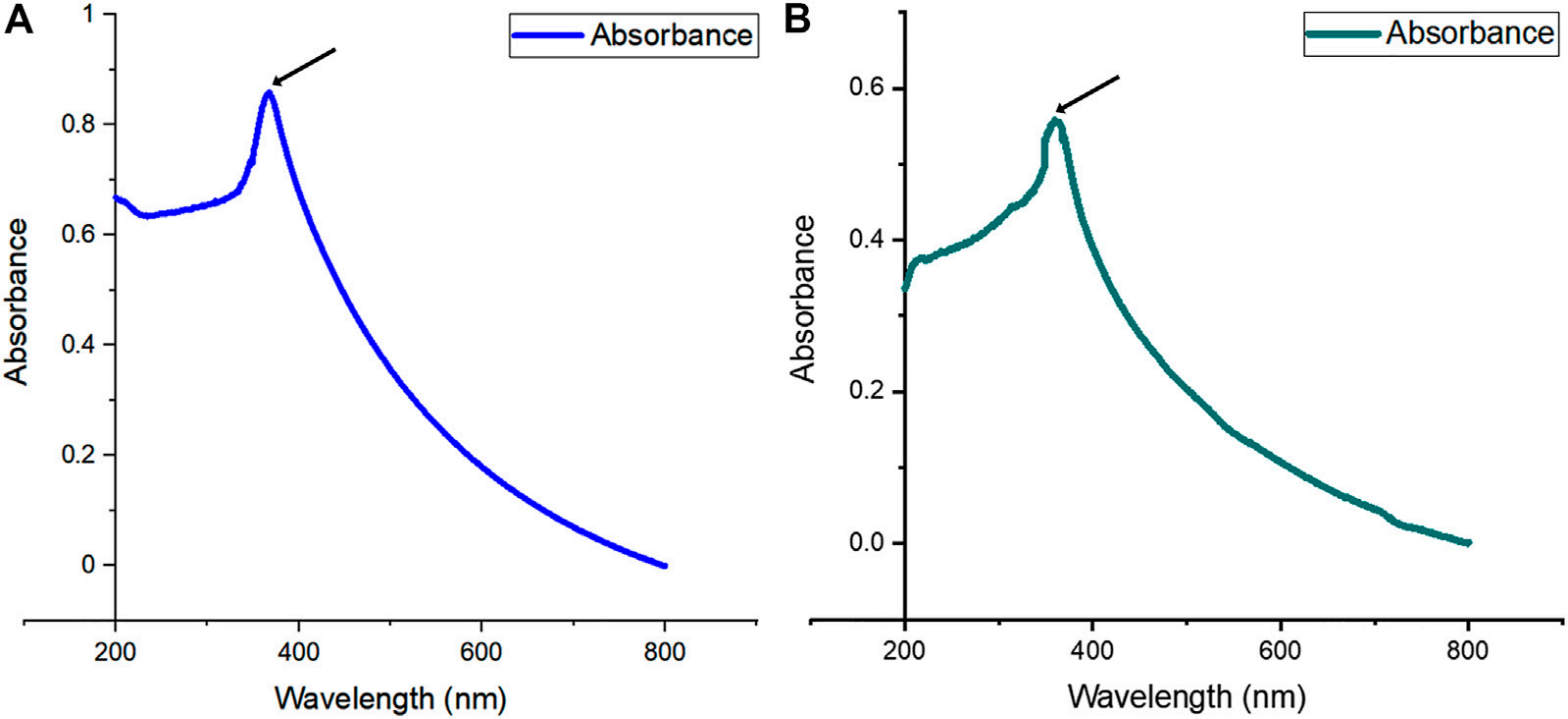
UV-visible (UV-vis) absorption spectra of biogenic ZnO-NPs (from 200 to 800-nm wavelength). **(A)** Ethanol extract of *S. chirayita* leaves and **(B)** methanol extract of *S. chirayita* leaves. Analysis was done using Origin software.

UV-vis spectrum characterization of ZnO-NPs was done at room temperature. The analysis was done by scanning the samples at an absorption spectrum range from 200 to 800 nm, and the absorbance peak (*λ* max value) was observed at 368 and 364 nm for ethanol and methanol leaf extracts of *S. chirayita*, respectively (Figure 2). This is in accordance with previous studies reporting that ZnO-NPs showed a maximum absorbance wavelength with in the range of 300–400 nm (Nethravathi et al., 2015; Jamdagni et al., 2018; Kuruppu et al., 2020; Wijesinghe et al., 2021).

#### 3.2.2 Dynamic Light Scattering

DLS was performed to find out the size distribution pattern of ZnO-NPs. It was diluted in Milli-Q water (1:10) and then passed to a cuvette for analysis. The DLS analysis showed the size distribution of nanoparticles, with an average hydrodynamic size of 285 and 209.9 nm and a low polydispersity index of 0.274 and 0.185 for ZnO-NPs of ethanol and methanol leaf extracts of *S. chirayita*, respectively, indicating good uniformity of ZnO-NPs (Supplementary Figure S1).

#### 3.2.3 Fourier Transform Infrared Spectroscopy

**FIGURE 3:**
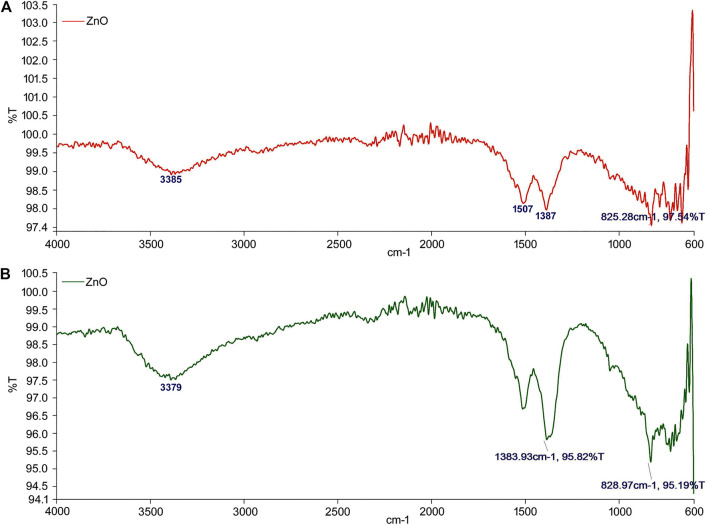
Fourier transform infrared spectroscopy analysis of biogenic ZnO-NPs. **(A)** Ethanol extract of *S. chirayita* leaves and **(B)** methanol extract of *S. chirayita* leaves.

FTIR studies were carried out in order to ascertain the phyto-chemical interactions present between the plant constituents/functional groups and the nanoparticle. This interaction helped in the reduction and stabilization of the synthesized ZnO-NPs. The observed FTIR results of ZnO-NPs were in accordance with previous studies (Awwad et al., 2013; Cheng et al., 2020). The FTIR analysis of synthesized ZnO-NPs from the ethanol extract of *S. chirayita* leaf showed wide peaks present at 3,385 cm^−1^, which reflects the presence of O–H stretching vibrations corresponding to an alcohol compound group, whereas 1,507, 1,387, 829, and 719 cm^−1^ peaks are due to the presence of N–O stretching, C–H bending, C=C bending, and again C=C bending, which correspond to nitro compound, aldehyde, alkene, and alkene, respectively (Figure 3A). In the same procedure, the analysis of synthesized ZnO-NP methanol extract of *S. chirayita* leaves showed wide peaks present at 3,379 cm^−1^, which reflects the presence of O–H stretching that corresponds to an alcohol group, whereas 1,506, 1,379, 835, and 736 cm^−1^ peaks are due to the presence of N–O stretching, C–H bending, C=C bending, and again C=C bending, which correspond to nitro compound, alkane, alkene, and alkene, respectively (Figure 3B).

#### 3.2.4 X-Ray Diffraction

**FIGURE 4:**
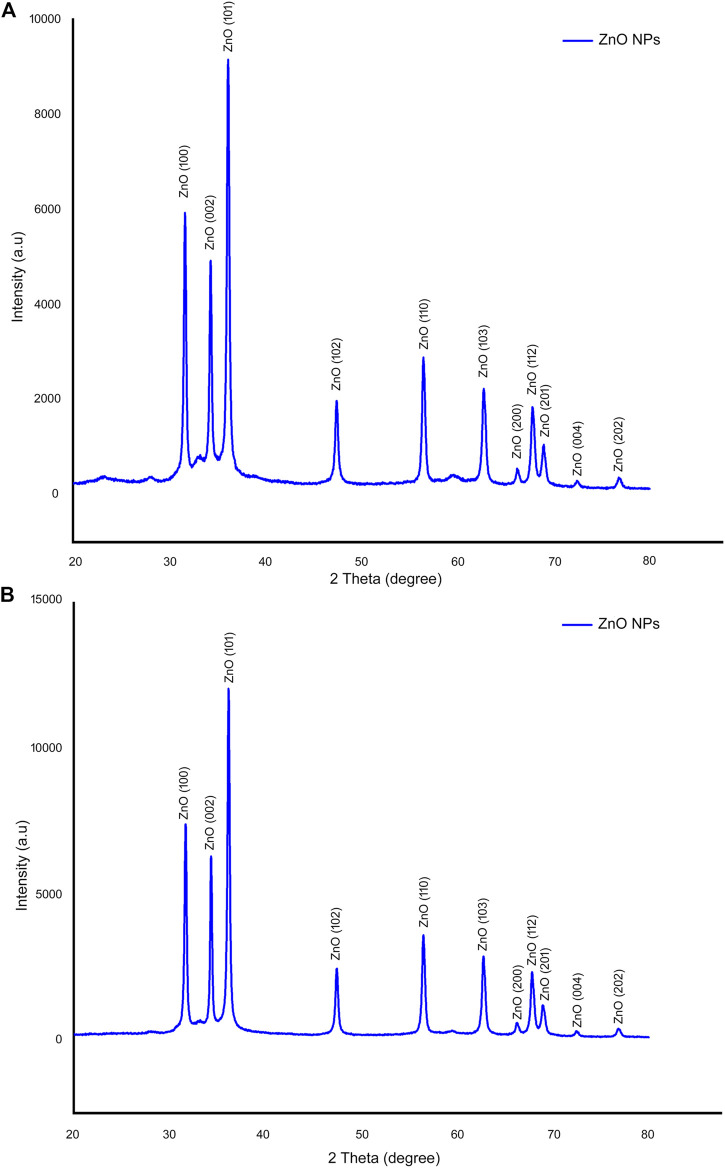
X-ray diffraction (XRD) analysis of biogenic ZnO-NPs. **(A)** Ethanol extract of *S. chirayita* leaves and **(B)** methanol extract of *S. chirayita* leaves. Analysis was done using Origin software.

The biogenic ZnO-NP’s XRD spectrum was analyzed using an X-ray diffraction, resulting in various crystal planes. The crystalline peaks positioned at (2*θ*) peak angles of 31.65°, 34.30°, 36.14°, 47.42°, 56.44°, 62.74°, 66.20°, 67.76°, 68.99°, 72.40°, and, 76.77° corresponding to the reflections from (100), (002), (101), (102), (110), (103), (200), (112), (201), (004), to (202) crystal planes were observed for ZnO-NPs from ethanol extract of *S. chirayita* leaves. Meanwhile, for ZnO-NPs from methanol extract of *S. chirayita* leaves, crystalline peaks positioned at (2*θ*) peak angles of 31.67°, 34.32°, 36.14°, 47.44°, 56.48°, 62.76°, 66.18°, 67.80°, 69.01°, 72.42°, and 76.83° correspond to the reflections from (100), (002), (101), (102), (110), (103), (200), (112), (201), (004), to (202) crystal planes, respectively. The results revealed that ZnO has a hexagonal structure and that the products have excellent crystallinity. These planes are a good fit for the quartzite ZnO hexagonal structure, and the same procedure was also followed by previous studies (Nadia et al., 2019). The ZnO-NPs of both the extracts have been keenly revealed as hexagonal wurtzite with lattice constants *a* = *b* = 0.324 nm and *c* = 0.521 nm (JPCDS card number: 36-1451). The average crystalline sizes of the ZnO-NPs were 24.67 and 22.95 nm for ethanol and methanol extracts of *S. chirayita* leaves, respectively. The XRD result illustrated that using different solvents for extraction of *S. chirayita* affected the size of the nanoparticles. It also proves that the produced nanopowder was devoid of contaminants because it lacks any other XRD peaks other than ZnO peaks. The average size of ZnO-NPs was calculated from the highest intensity peaks (101) using the Debye-Scherrer equation (Cao et al., 2011) (Figure 4).

#### 3.2.5 Scanning Electron Microscopy

**FIGURE 5:**
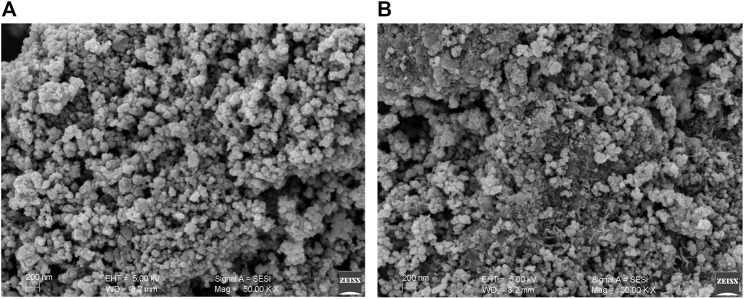
Scanning electron microscopy (SEM) image of biogenic ZnO-NPs. **(A)** Ethanol extract of *S. chirayita* leaves and **(B)** methanol extract of *S. chirayita* leaves.

SEM was applied to survey the morphology of ZnO-NPs. SEM images of ZnO-NPs synthesized from ethanol and methanol extracts of *S. chirayita* leaves were generated. Topographic view showed that the ZnO-NPs are spherical in shape and are clustered nanocrystallines (Figure 5). Similar outcomes for SEM analysis were disclosed by other studies (Naseer et al., 2020; Pan and Zhong, 2016). The aggregation of ZnO-NPs is expected due to the fact that nanoparticles have a larger surface area and adhere to one another more firmly. As a result, environmental variables have a significant impact on the stability of NP_S_ and their aggregation (Sundrarajan et al., 2015).

#### 3.2.6 Transmission Electron Microscopy

**FIGURE 6:**
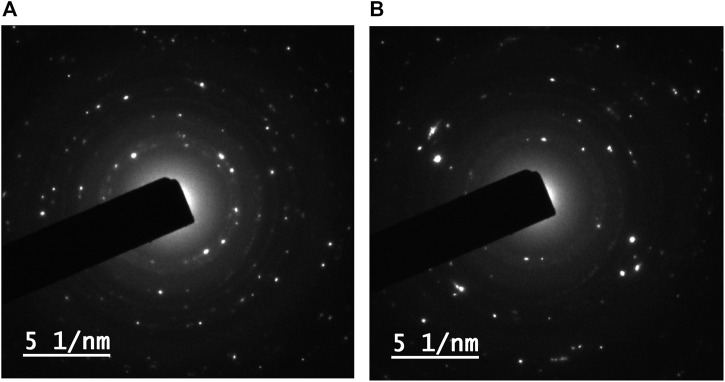
Transmission electron microscopy (TEM) image of biogenic ZnO-NPs. **(A)** Ethanol extract of *S. chirayita* leaves and **(B)** methanol extract of *S. chirayita* leaves.

The shape and particle size distribution of ZnO-NPs were further examined using HR-TEM for additional characterization. The spherical form of ZnO-NPs in TEM images with an estimated average size of 32.11 ± 7.659 and 33.27 ± 5.851 nm for synthesized ZnO-NPs from ethanol and methanol extracts of *S. chirayita* leaves, respectively (Figure 6), demonstrates that the nanoparticles have a good crystallization.

### 3.3 Cytotoxic Activities

**FIGURE 7:**
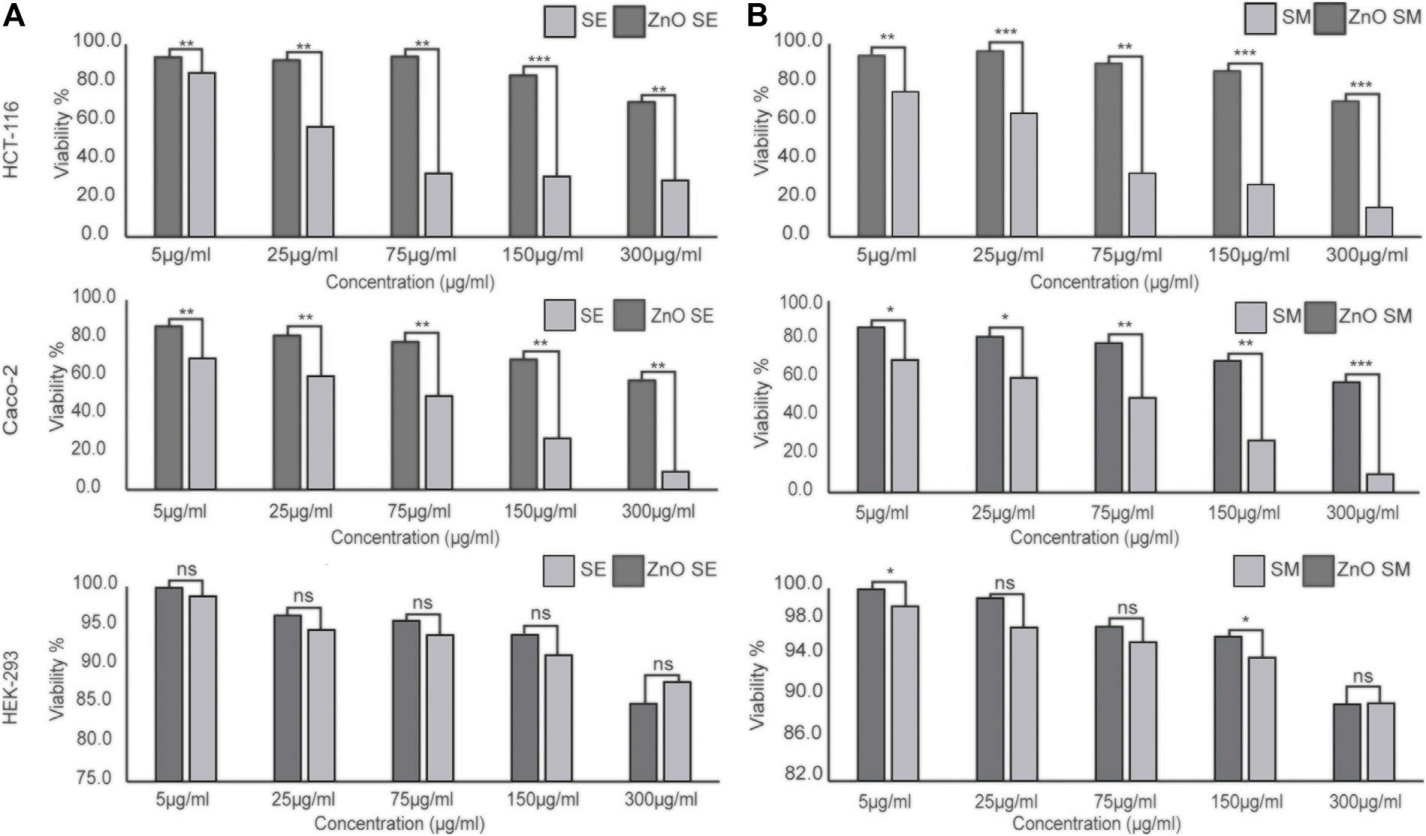
In vitro cytotoxic efficacy of crude extract and green ZnO-NPs synthesized from ethanol **(A)** and methanol extracts **(B)** of *S. chirayita* leaves. Colorectal cancer (CRC) cells (HCT-116 and Caco-2) and control cells (HEK-293) were treated with SE, SM, ZnO SE, and ZnO SM. The results are expressed as cell viability percentage (%) in comparison between treatments of crude extract and corresponding ZnO-NPs conjugated with the phyto-constituents of the plant. The data represent the mean values ± SD of three independent experiments performed in triplicate. Statistical values were calculated using ANOVA: single factor. ^*^ *p* ≤ 0.05, ^**^ *p* ≤ 0.01, ^***^ *p* ≤ 0.001, nsP > 0.05. SE, ethanol extract of *S. chirayita* leaves; SM, methanol extract of *S. chirayita* leaves; ZnO SE, biogenic ZnO-NPs conjugated with ethanol extract of *S. chirayita* leaves; ZnO SM, biogenic ZnO-NPs conjugated with methanol extract of *S. chirayita* leaves.

In this study, the cytotoxic effect of plant extracts and synthesized ZnO-NPs was evaluated against two colorectal cancer cell lines, HCT-116 and Caco-2, and a control cell line, HEK-293, using MTT assay. MTT assay is considered as a highly accurate and sensitive colorimetric method to check the cell viability after exposure to different therapeutic substances. It is dependent on the ability of succinate dehydrogenase mitochondrial enzyme to change the tetrazolium yellow dye to formazan crystals, which is directly proportional to the cell viability and assayed as optical density (Rai et al., 2018). Data analysis revealed that the viability of the treated cell lines was dose dependent; as the NP concentration increased, the viability was decreased. This finding is constant with other previous studies (Rajakumar et al., 2018). The IC_50_ values of ethanolic and methanolic extract ZnO-NPs for HCT-116, Caco-2, and HEK-293 were 34.356 ± 2.71 and 32.856 ± 2.99 μg/ml, 52.15 ± 8.23 and 63.1 ± 12.09 μg/ml, and 582.84 ± 5.26 and 615.35 ± 4.74 μg/ml, respectively (Figures 7A,B). According to these results, it can be inferred that the synthesized nanoparticles from ethanol and methanol crude extracts of *S. chirayita* leaves (ZnO SM and ZnO SE) showed cytotoxicity on cancer cells with respect to the crude extracts SM and SE, and the NPs exhibit more cytotoxic potential on cancer cells (HCT-116, and Caco-2) when compared to control cells (HEK-293) (Figures 7A,B).

### 3.4 Acridine Orange Staining

**FIGURE 8:**
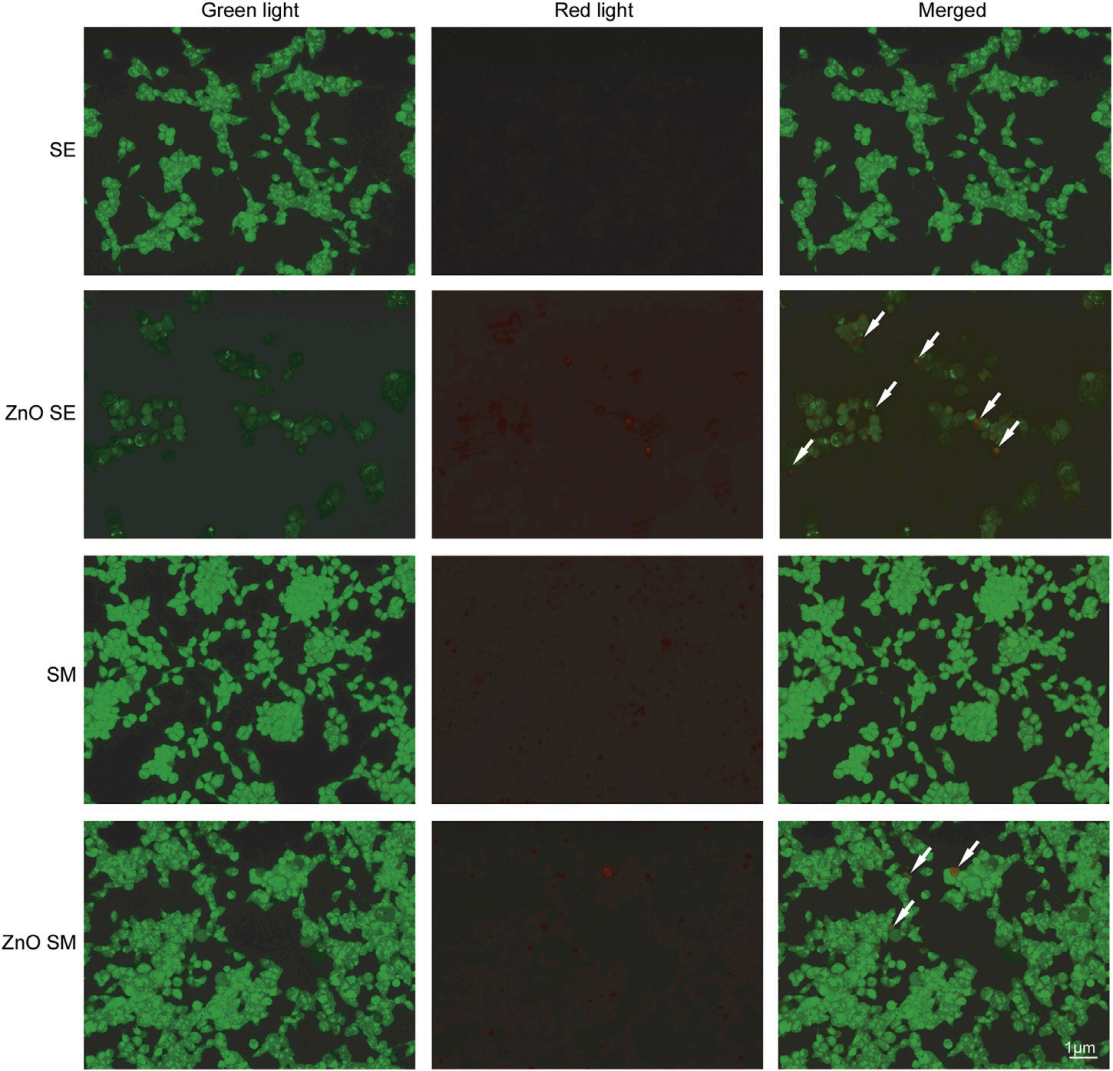
Acridine orange (AO) staining. Acridine orange staining of Caco-2 cells to detect apoptosis. Live cells are uniformly green, whereas apoptotic cells are stained orange-red due to chromatin condensation and loss of membrane integrity. Pictures were taken at magnification of ×40.

Caco-2 cells treated with SE and ZnO SE of *S. chirayita* leaves were stained with AO. The result was examined under a fluorescence microscope. The result showed that no significant apoptosis was detected in cells treated with SE of *S. chirayita* leaves, whereas cells treated with biogenic ZnO SE of *S. chirayita* leaves revealed apoptosis (orange/red spots) characterized by chromatin condensation (Figure 8). As reported by various authors, apoptotic signs, plasma membrane blebbing, and crescent-shaped or granular yellow-green AO nuclear staining were observed (Hsin et al., 2008).

### 3.5 Gene Expression Analysis of Cancer Markers

**FIGURE 9:**
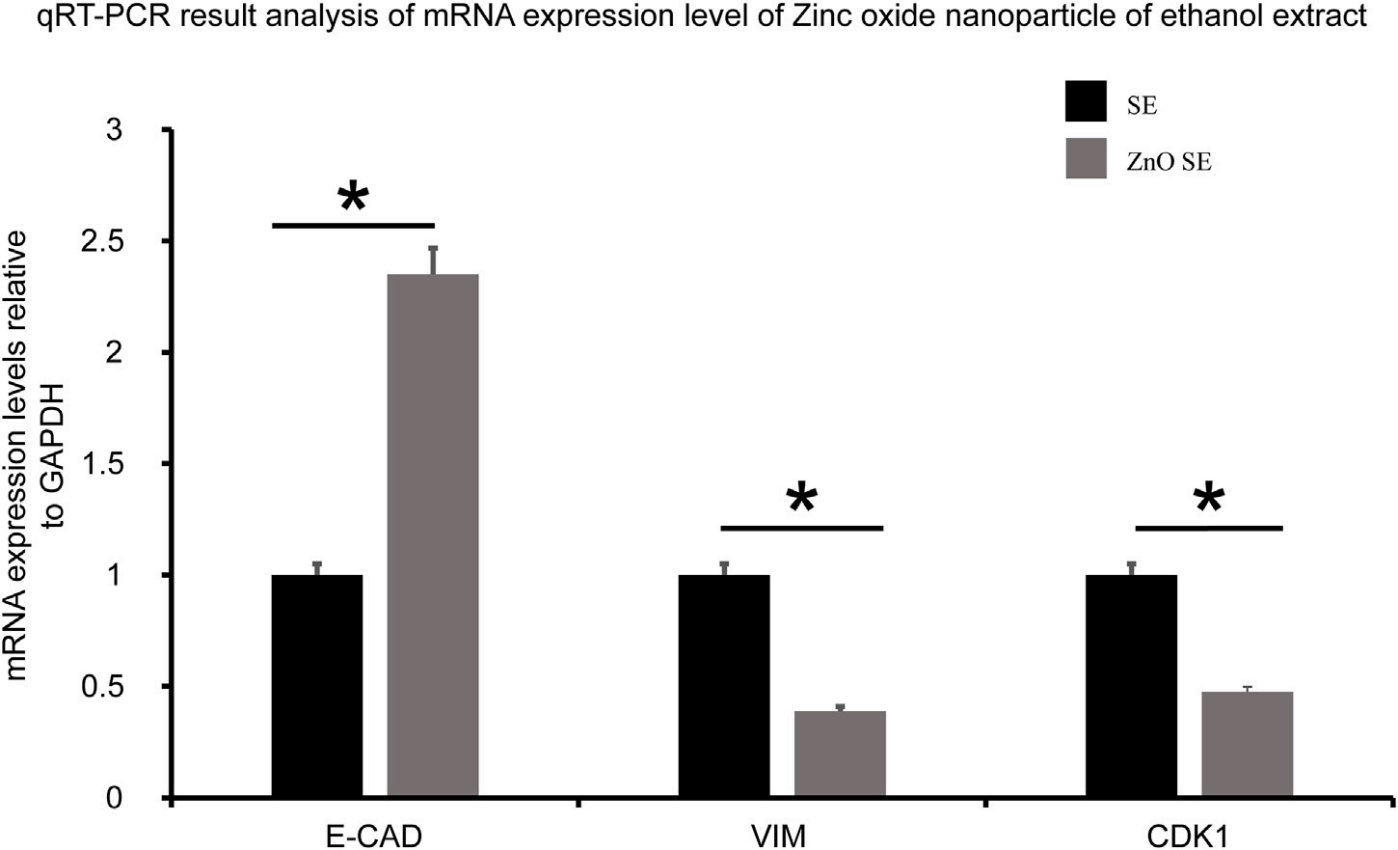
Quantitative real-time PCR (qRT-PCR) analysis. The expression level of CDK1 and vimentin in Caco-2 cell lines treated with ZnO SE reduced by 0.525 and 0.62-fold, respectively (with *p*-Value = 0.013 and 0.041), whereas the expression level of E-cadherin increased 2.35-fold (*p*-Value = 0.049) as compared to cells treated with SE.

qRT-PCR was used to examine the mRNA levels of some cancer markers such as cell cycle marker (CDK1), epithelial marker (E-cadherin), and mesenchymal marker (vimentin) in Caco-2 cells treated with SE and ZnO SE. Cells treated with ZnO SE had shown a distinct effect on the expression level of CDK1, E-cadherin, and vimentin. The expression level of CDK1 and vimentin was reduced by 0.525 and 0.62-fold, respectively (with *p*-values 0.013 and 0.041, respectively), whereas the expression level of E-cadherin increased 2.35-fold (*p*-value 0.049) as compared to the SE treatment (Figure 9).

## 4 Discussion

In this work, ZnO-NPs were synthesized using two separate solvents ethanol (80%) and methanol (90%) extracts of *S. chirayita* leaves. The phyto-chemical GC-MS analysis of ethanol extract from the leaves of *S. chirayita* revealed that only 21 peaks were observed, but the methanol extract reveals 35 peaks. Related studies had shown that the methanolic extract of *S. chirayita* showed only 15 peaks *via* HPLC (Wawrosch et al., 2005). Aleem and Kabir (2018) also summarized around 21 chemical constituents of *S. chirayita* and its broad biological activities (Karimzadeh et al., 2020). Active analytes with a high polyphenolic concentration can bind metal ions and help with the capping and reduction of the ZnO-NPs. UV-visible spectrum displayed a distinct peak around 364 and 368 nm for ethanol and methanol extract ZnO-NPs, respectively. There have been no more significant peak shifts observed, which is particular for ZnO-NPs. This is in accordance with previous studies reporting that ZnO-NPs show a maximum absorbance wavelength within the range of 300–400 nm (Nethravathi et al., 2015; Jamdagni et al., 2018; Kuruppu et al., 2020; Wijesinghe et al., 2021).

The synthesis method was found to be efficient, as evidenced by the generation of single-crystalline ZnO-NPs with a hexagonal wurtzite structure, as determined by XRD. The average crystallite size of the nanoparticles was 24.67 and 22.95 nm for ZnO-NPs synthesized by ethanol and methanol solvents, respectively, exhibiting spherical-like structures. The ZnO-NPs’ XRD spectrum was detected using X-ray diffraction, resulting in various crystal planes. The crystalline peaks positioned at (2*θ*) angles of 31.65°, 34.30°, 36.14°, 47.42°, 56.44°, 62.74°, 66.20°, 67.76°, 68.99°, 72.40°, and 76.77° corresponding to the reflections from (100), (002), (101), (102), (110), (103), (200), (112), (201), (004), to (202) crystal planes were observed for ZnO-NPs from ethanol extract of *S. chirayita* leaves. Meanwhile, for ZnO-NPs from methanol extract of *S. chirayita* leaves, crystalline peaks positioned at (2*θ*) peak angles of 31.67°, 34.32°, 36.14°, 47.44°, 56.48°, 62.76°, 66.18°, 67.80°, 69.01°, 72.42°, and 76.83° correspond to the reflections from (100), (002), (101), (102), (110), (103), (200), (112), (201), (004), to (202) crystal planes, respectively. The result showed that the synthesized ZnO-NPs have a hexagonal form and have excellent crystallinity. These planes fit well with previous studies (Bhatte et al., 2012; Nadia et al., 2019; Naseer et al., 2020) as well as JCPDS card no. 36-1451.

FTIR spectroscopy was used to determine the functional groups in the production of ZnO-NPs. The wide peaks at 3,385 and 3,379 cm^−1^ reflect the presence of O–H stretching corresponding to an alcohol compound group in the ZnO-NPs from ethanol and methanol extracts of *S. chirayita* leaves, respectively. These results are inconsistent with previous studies (Cheng et al., 2020). According to the FTIR analysis, the synthesized ZnO-NPs are conjugated with different phyto-chemicals, which act as capping and stabilizing agents for the synthesis of ZnO-NPs.

For the ZnO-NPs synthesized from ethanol and methanol extracts of *S. chirayita* leaves, DLS analysis revealed a size distribution of particles with an average hydrodynamic size of 285 and 209.9 nm, respectively. Similar previous studies showed that the average size from the DLS analysis was larger than that from XRD and TEM, and this happened due to the polydisperse nature of NPs (Jamdagni et al., 2018). TEM analysis showed that the major series of particle size was between 19.8 and 23.4 nm, and SEM analysis showed that the ZnO-NPs are spherical in shape and are clusters of nanocrystallites, which is supported by previous similar findings too (Naseer et al., 2020; Pan and Zhong, 2016).

Data analysis for cytotoxicity effect revealed that the viability of treated cell lines was dose dependent; as the concentration of ZnO-NPs increased, the viability decreased (Sanaeimehr et al., 2018). Cytotoxicity activities of ethanol and methanol crude extracts of *S. chirayita* leaves (SE and SM) and synthesized nanoparticles (ZnO SE and ZnO SM) were analyzed by MTT assay. In this study, we showed that the biogenic ZnO-NPs have cytotoxic activities against colorectal cancer cell lines (HCT-116 and Caco-2) but insignificant activities against the control cell line (HEK-293). Both the ZnO SE and ZnO SM showed significant cytotoxic activities in a concentration-dependent manner (5–300 μg/ml). Our findings showed that ZnO-NPs have more cytotoxic effects compared to the corresponding crude extract of *S. chirayita* leaves. The IC_50_ values of ethanolic and methanolic extract ZnO-NPs for HCT-116, Caco-2, and HEK-293 were 34.356 ± 2.71 and 32.856 ± 2.99 μg/ml, 52.15 ± 8.23 and 63.1 ± 12.09 μg/ml, and 582.84 ± 5.26 and 615.35 ± 4.74 μg/ml, respectively. Similarly, other studies also showed that biologically synthesized ZnO-NPs had anticancer activities in HCT-116 cell lines (Majeed et al., 2019). It was shown that Caco-2 cell lines treated with green-synthesized ZnO-NPs displayed cytotoxic activities with an IC_50_ value of 9.95 ppm, after 48 h of treatment (El-Belely et al., 2021). Similarly, Kang et al. (2013) showed 15.55 ± 1.19 μg/ml, 22.84 ± 1.36 μg/ml, and 18.57 ± 1.27 μg/ml IC_50_ values for three different sizes of green-synthesized ZnO-NPs—26, 62, and 90 nm, respectively. Green-synthesized ZnO-NPs from *D. tortuosa* aqueous extract showed a profound selective cytotoxic effect on the Caco-2 and A549 cancer cell lines (Selim et al., 2020). Sanaeimehr et al. (2018) showed that green-synthesized zinc oxide nanoparticles using *Sargassum muticum* algae extract have a cytotoxic activity on HepG2 cell lines at an IC_50_ value of 150 μg/ml after 48 h of exposure time. Biogenic ZnO-NPs were found to have a cytotoxic effect against both HCT-116 and Caco-2 cancer cell lines but a reduced cytotoxic activity in normal human HEK-293 cell lines. The same result was demonstrated in various studies (Wahab et al., 2011; Shandiz et al., 2021).

AO staining showed that no significant apoptosis was detected in cells treated with SE, whereas cells treated with ZnO SE revealed apoptotic signs, plasma membrane blebbing, and crescent-shaped or granular yellow-green AO nuclear staining. Earlier, it has been shown that ZnO-NPs induced apoptosis as well as oxidative stress *in vitro* and *in vivo* (Verma et al., 2017; Das et al., 2019). This study is in line with the previous research done by Kang et al. (2013). In their paper, they demonstrated that AO analysis of Caco-2 cells treated with different doses of ZnO-NPs showed a significant reduction in viable cells and an increase in early apoptotic cells, late apoptotic cells, and necrotic cells (Kang et al., 2013).

qRT-PCR analysis of Caco-2 cell lines treated with ZnO SE showed a significantly reduced expression level of CDK1 and vimentin by 0.525 and 0.62-fold, respectively, whereas the expression level of E-cadherin increased 2.35-fold as compared to SE-treated cells. Cells treated with biogenic ZnO-NPs showed a reduced level of the expression of oncogene cancer markers, revealing its anticancer activities (Liu et al., 2013; Zhu et al., 2015).

A study done by Saha et al. (2004) showed that significant anticancer activities were observed after both the crude and purified *S. chirayita* extracts significantly inhibited cell proliferation and induced apoptosis. In summary, it could be concluded that the solvents we used for the extraction of plant phyto-constituents affect the application of the synthesized ZnO-NPs on cells. Biogenic ZnO-NPs are eco-friendly, cost-effective, and less toxic. This offers promising alternatives to current chemotherapy, but further biomolecular tests and mechanism-of-action and *in vivo* studies should be evaluated for further use.

## 5 Conclusion

In summary, the findings show that the biogenic synthesized ZnO-NPs from the extract of *S. chirayita* leaves are pure-standard NPs and spherical in shape, with anticancer activities. These were proven by characterization of the biogenic ZnO-NPs through UV-vis, FTIR, XRD, SEM, and TEM. X-ray diffraction analysis ratified the formation of zinc oxide hexagonal wurtzite structure with spherical shape. Their anticancer activities were also proved by performing the cytotoxic assay, AO staining, and qRT-PCR. The synthesized nanoparticles have the potential to become alternative anti-cancer treatments, but further *in vitro* and *in vivo* studies are needed.

## Data Availability

The original contributions presented in the study are included in the article/Supplementary Material; further inquiries can be directed to the corresponding author.
