# Supplementary figures and images for: Cytotoxic Potential of Biogenic Zinc Oxide Nanoparticles Synthesized From Swertia chirayita Leaf Extract on Colorectal Cancer Cells
Source: Front Bioeng Biotechnol. 2021 Dec 15;9:788527. doi: 10.3389/fbioe.2021.788527 (PMC8714927; doi:10.3389/fbioe.2021.788527)

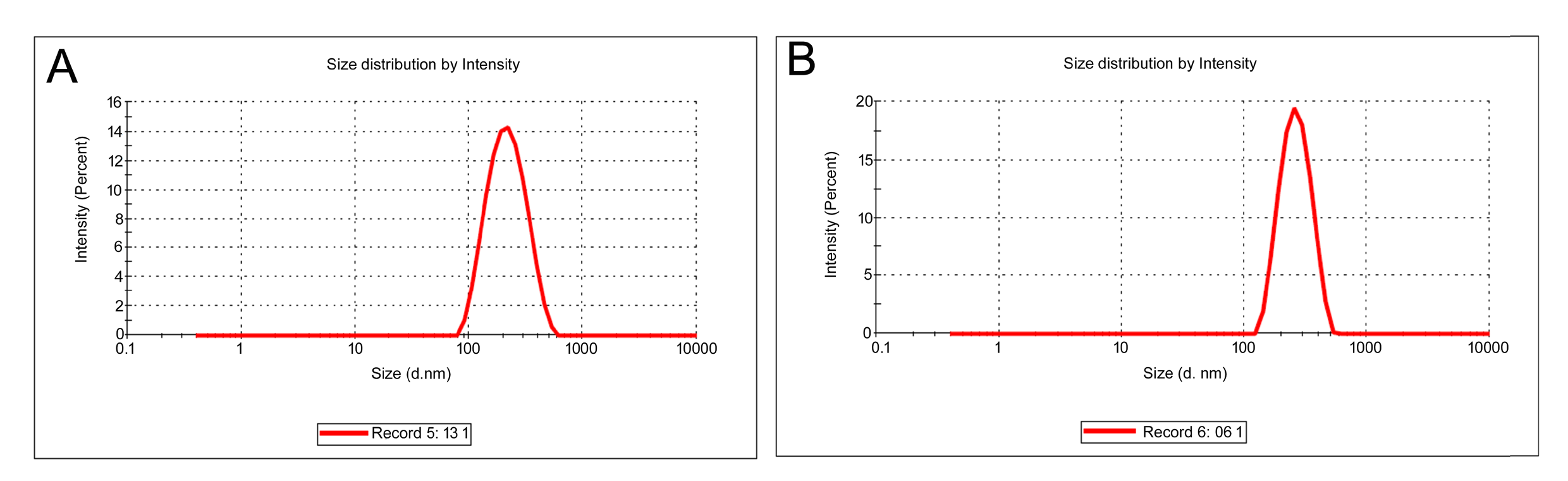

Supplement: Supplementary file 1 [file Image1.TIF]
