# Supplementary material for: Cytotoxic Potential of Biogenic Zinc Oxide Nanoparticles Synthesized From Swertia chirayita Leaf Extract on Colorectal Cancer Cells
Source: Front Bioeng Biotechnol. 2021 Dec 15;9:788527. doi: 10.3389/fbioe.2021.788527 (PMC8714927; doi:10.3389/fbioe.2021.788527)
Supplement: Supplementary file 2 [file DataSheet1.docx]

### Cytotoxic potential of Biogenic Zinc oxide nanoparticles synthesized from *Swertia chirayita* leaf extract on Colorectal cancer cells

### Hadgu Mendefro Berehu^1^, Anupriya S^1^, Imran Khan^1^, Rajasree Chakraborty^1^, Kousalya Lavudi^1^, Josthna P^2^, Bibhshee Mohapatra^1^, Amrita Mishra^1^, Srinivas Patnaik^1^*

^1^Disease Biology Laboratory, School of Biotechnology KIIT Deemed to be University, Bhubaneswar, Odisha-751024, India.

^2^Department of Biotechnology, Sri Padmavati Mahila Visvavidyalam, Tirupati, 517502, A.P, India

*Correspondence to: **Srinivas Patnaik**, Associate Professor, School of Biotechnology, KIIT Deemed to be University, Bhubaneswar, Odisha, India. Email: [Srinivas.patnaik@kiitbiotech.ac.in](mailto:Srinivas.patnaik@kiitbiotech.ac.in)

**Supplementary Table S1**: List of phytochemical compounds detected in the ethanol leaf extract of *S. chirayita* using GC-MS.

| **Peak #** | **Compound Name** | **PubChem CID** | **Molecular formula** |
| --- | --- | --- | --- |
| **1** | 14-Oxa-1,11-diazatetracyclo[7.4.1.0(2,7) | 133974 | [C_20_H_21_N_3_O_9_](https://pubchem.ncbi.nlm.nih.gov/#query=C20H21N3O9) |
| **2** | Diethoxymethyl acetate | 84166 | [C_7_H_14_O_4_](https://pubchem.ncbi.nlm.nih.gov/#query=C7H14O4) |
| **3** | 1-Undecene, 4-methyl | 522551 | [C_12_H_24_](https://pubchem.ncbi.nlm.nih.gov/#query=C12H24) |
| **4** | Phenol, 2,4-bis(1,1-dimethylethyl) | 93344 | [C_22_H_30_O](https://pubchem.ncbi.nlm.nih.gov/#query=C22H30O) |
| **5** | 3,4-diethylphthalate | 22026949 | [C_12_H_12_O_4_^-2^](https://pubchem.ncbi.nlm.nih.gov/#query=C12H12O4-2) |
| **6** | Pentadecane | 12391 | C_15_H_32_ |
| **7** | Cyclohexanecarboxylic acid, 2-ethylhexyl | 85993 | [C_15_H_28_O_2_](https://pubchem.ncbi.nlm.nih.gov/#query=C15H28O2) |
| **8** | Carbonic acid, butyl 3-methylphenyl este | 767 | [CH_2_O_3_](https://pubchem.ncbi.nlm.nih.gov/#query=CH2O3) |
| **9** | Nonadecane | 12401 | [C_19_H_40_](https://pubchem.ncbi.nlm.nih.gov/#query=C19H40) |
| **10** | 3,7,11,15-Tetramethyl-2-hexadecen-1-ol | 5366244 | [C_20_H_40_O](https://pubchem.ncbi.nlm.nih.gov/#query=C20H40O) |
| **11** | 8-Hexadecenal, 14-methyl-, (Z) | 5364688 | [C_17_H_32_O](https://pubchem.ncbi.nlm.nih.gov/#query=C17H32O) |
| **12** | Bicyclo[10.8.0]eicosane, €- | 534682 | [C_20_H_38_](https://pubchem.ncbi.nlm.nih.gov/#query=C20H38) |
| **13** | 1,4-Eicosadiene | 5365774 | [C_20_H_38_](https://pubchem.ncbi.nlm.nih.gov/#query=C20H38) |
| **14** | 7,9-Di-tert-butyl-1-oxaspiro(4,5)deca-6 | 545303 | C_17_H_24_O_3_ |
| **15** | Phthalic acid, 8-chlorooctyl isobutyl es | 1017 | [C_8_H_6_O_4_](https://pubchem.ncbi.nlm.nih.gov/#query=C8H6O4) or C_6_H_4_(COOH)  or  H_2_C_8_H_4_O_4_ |
| **16** | 2-Ethylamino-3-methyl(phenyl)amino-1,4-n | 627860 | [C_19_H_18_N_2_O_2_](https://pubchem.ncbi.nlm.nih.gov/#query=C19H18N2O2) |
| **17** | Phytol | 5280435 | [C_20_H_40_O](https://pubchem.ncbi.nlm.nih.gov/#query=C20H40O) |
| **18** | 9,12-Octadecadienoic acid (Z,Z)- | 5280450 | [C_18_H_32_O_2_](https://pubchem.ncbi.nlm.nih.gov/#query=C18H32O2) |
| **19** | 7,10,13-Hexadecatrienoic acid, methyles | 5367325 | [C_17_H_28_O_2_](https://pubchem.ncbi.nlm.nih.gov/#query=C17H28O2) |
| **20** | Octadecanoic acid, 2-(2-hydroxyethoxy)et | 5281 | [C_18_H_36_O_2_](https://pubchem.ncbi.nlm.nih.gov/#query=C18H36O2) or CH_3_(CH_2_)_16_COOH |
| **21** | Tritriacontane | 12411 | [C_33_H_68_](https://pubchem.ncbi.nlm.nih.gov/#query=C33H68) |

**Supplementary Table S2**: List of phytochemical compounds detected in the methanol leaf extract of *S. chirayita* using GC-MS.

| **Peak #** | **Compound Name** | **PubChem CID** | **Molecular formula** |
| --- | --- | --- | --- |
| **1** | Cyclobutane, 2-ethyl-1-methyl-3-propyl | 550796 | [C_10_H_20_](https://pubchem.ncbi.nlm.nih.gov/#query=C10H20) |
| **2** | 4-tert-Butoxystyrene | 577531 | [C_12_H_16_O](https://pubchem.ncbi.nlm.nih.gov/#query=C12H16O) |
| **3** | 4-Hydroxy-2-methylacetophenone | 70133 | [C_9_H_10_O_2_](https://pubchem.ncbi.nlm.nih.gov/#query=C9H10O2) |
| **4** | (5R,8R,8aS)-8-Methyl-5-propyl-1,2,3,5,8, | 74340628 | [C_12_H_21_N](https://pubchem.ncbi.nlm.nih.gov/#query=C12H21N) |
| **5** | Phenol,2,4-bis(1,1-dimethylethyl)- | 69224 | [C_15_H_24_O](https://pubchem.ncbi.nlm.nih.gov/#query=C15H24O) |
| **6** | 2(4H)-Benzofuranone, 5,6,7,7a-tetrahydro | 11789269 | [C_8_H_10_O_2_](https://pubchem.ncbi.nlm.nih.gov/#query=C8H10O2) |
| **7** | Phthalic acid, 2-cyclohexylethyl isobuty | - | - |
| **8** | Cyclohexanecarboxylic acid, 2-ethylhexyl | 85993 | [C_15_H_28_O_2_](https://pubchem.ncbi.nlm.nih.gov/#query=C15H28O2) |
| **9** | trans-Z-.alpha.-Bisabolene epoxide | 5363099 | C_15_H_24_O |
| **10** | 1,5,9,9-Tetramethyl-2-methylene-spiro[3. | 577131 | [C_14_H_22_](https://pubchem.ncbi.nlm.nih.gov/#query=C14H22) |
| **11** | Benzophenone | 3102 | [C_13_H_10_O](https://pubchem.ncbi.nlm.nih.gov/#query=C13H10O) or C_6_H_5_COC_6_H_5_ |
| **12** | 3,7-Cycloundecadien-1-ol, 1,5,5,8-tetram | 5367678 | [C_15_H_26_O](https://pubchem.ncbi.nlm.nih.gov/#query=C15H26O) |
| **13** | 8-Pentadecanone | 13162 | [C_15_H_30_O](https://pubchem.ncbi.nlm.nih.gov/#query=C15H30O) |
| **14** | 4-((1E)-3-Hydroxy-1-propenyl)-2-methoxyp | - | - |
| **15** | Tetradecanoic acid | 11005 | [C_14_H_28_O_2_](https://pubchem.ncbi.nlm.nih.gov/#query=C14H28O2) |
| **16** | Acetic acid, 2-(2,2,6-trimethyl-7-oxa-bi | 176 | [C_2_H_4_O_2_](https://pubchem.ncbi.nlm.nih.gov/#query=C2H4O2) or CH_3_COOH |
| **17** | 1-Eicosanol | 12404 | [C_20_H_42_O](https://pubchem.ncbi.nlm.nih.gov/#query=C20H42O) |
| **18** | 3,7,11,15-Tetramethyl-2-hexadecen-1-ol | 5366244 | [C_20_H_40_O](https://pubchem.ncbi.nlm.nih.gov/#query=C20H40O) |
| **19** | 1,4-Eicosadiene | 5365774 | [C_20_H_38_](https://pubchem.ncbi.nlm.nih.gov/#query=C20H38) |
| **20** | Cyclopropanenonanoic acid, 2-[(2-butylcy | 544392 | [C_21_H_38_O_2_](https://pubchem.ncbi.nlm.nih.gov/#query=C21H38O2) |
| **21** | 8-Pentadecanone | 13162 | [C_15_H_30_O](https://pubchem.ncbi.nlm.nih.gov/#query=C15H30O) |
| **22** | 1,4-Eicosadiene | 549368 | [C_20_H_38_](https://pubchem.ncbi.nlm.nih.gov/#query=C20H38) |
| **23** | [3,3-Dimethyl-2-(3-methylbuta-1,3-dienyl | 572864 | [C_14_H_24_O](https://pubchem.ncbi.nlm.nih.gov/#query=C14H24O) |
| **24** | Hexadecanoic acid, 2-methyl | 117384 | [C_17_H_34_O_2_](https://pubchem.ncbi.nlm.nih.gov/#query=C17H34O2) |
| **25** | 1H-1,2,3-Triazole-4-carboxylic acid, 1-[ | 555194 | [C_9_H_7_N_3_O_2_](https://pubchem.ncbi.nlm.nih.gov/#query=C9H7N3O2) |
| **26** | n-Hexadecanoic acid | 985 | [C16H32O2](https://pubchem.ncbi.nlm.nih.gov/#query=C16H32O2) |
| **27** | Oleic Acid | 445639 | [C_18_H_34_O_2_](https://pubchem.ncbi.nlm.nih.gov/#query=C18H34O2) or C_8_H_17_CH=CH(CH_2_)_7_COOH |
| **28** | 10-Nonadecanone | 10441 | [C_19_H_38_O](https://pubchem.ncbi.nlm.nih.gov/#query=C19H38O) |
| **29** | Methyl 10-trans,12-cis-octadecadienoate | 5471014 | [C_19_H_34_O_2_](https://pubchem.ncbi.nlm.nih.gov/#query=C19H34O2) |
| **30** | 9-Octadecenoic acid (Z)-, 2-hydroxy-1-(h | 445639 | [C_18_H_34_O_2_](https://pubchem.ncbi.nlm.nih.gov/#query=C18H34O2) or C_8_H_17_CH=CH(CH_2_)_7_COOH |
| **31** | Phytol, acetate | 6428538 | [C_22_H_42_O_2_](https://pubchem.ncbi.nlm.nih.gov/#query=C22H42O2) |
| **32** | Methyl 8,11,14-heptadecatrienoate | 85978449 | [C18H30O2](https://pubchem.ncbi.nlm.nih.gov/#query=C18H30O2) |
| **33** | 9,12-Octadecadienoic acid (Z,Z)- | 5280450 | [C18H32O2](https://pubchem.ncbi.nlm.nih.gov/#query=C18H32O2) |
| **34** | Oleic Acid | 445639 | [C_18_H_34_O_2_](https://pubchem.ncbi.nlm.nih.gov/#query=C18H34O2) or C_8_H_17_CH=CH(CH_2_)_7_COOH |
| **35** | Acetic acid, 4,5-dihydroxy-10,13-dimethy | 537189 | [C21H32O5](https://pubchem.ncbi.nlm.nih.gov/#query=C21H32O5) |

**Supplementary Table S3**: List of sequence of primers used for qRT-PCR

| 1. NO | Primers used for qRT-PCR | Gene Forward (F) and Reverse (R) Primers | TM (^0^C) |
| --- | --- | --- | --- |
| 1 | GAPDH F: | 5’-GAGCGAGATCCCTCCAAAAT-3’ | 59.82 |
|  | GAPDH R: | 5’-GGCTGTTGTCATACTTCTCATG-3’ | 58.39 |
| 2 | CDK1 F: | 5’-TGGATTCTATCCCTCCTGGTC-3’ | 59.82 |
|  | CDK1 R: | 5’-CTCTGGCAAGGCCAAAATCAG-3’ | 59.82 |
| 3 | E-cadherin F: | 5’-TGGACCGAGAGAGTTTCCCT-3’ | 59.35 |
|  | E-cadherin R: | 5’-CAAAATCCAAGCCCGTGGTG-3’ | 59.35 |
| 4 | Vimentin F: | 5’-TCCACTGAGTACCGGAGACA-3’ | 59.35 |
|  | Vimentin R: | 5’-AGGTGACGAGCCATTTCCTC-3’ | 59.35 |
